# Supplementary figures and images for: A network-based analysis detects cocaine-induced changes in social interactions in Drosophila melanogaster
Source: PLoS One. 2023 Mar 23;18(3):e0275795. doi: 10.1371/journal.pone.0275795 (PMC10035901; doi:10.1371/journal.pone.0275795)

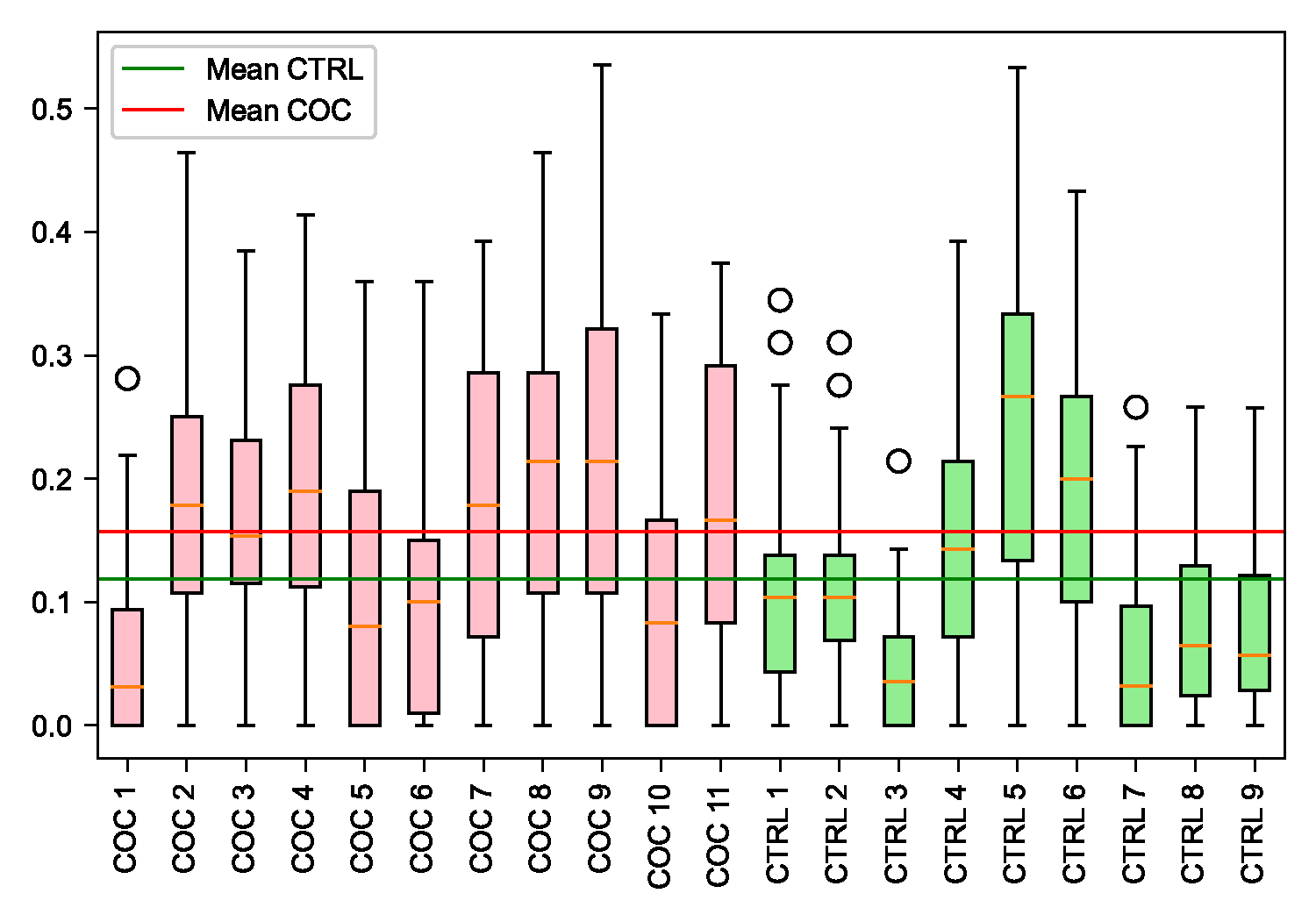

Supplement: S1 Fig — Box plot graph represents measures of Degree centrality measure distribution across networks in CTRL and COC populations. (TIF) [file pone.0275795.s002.tif]

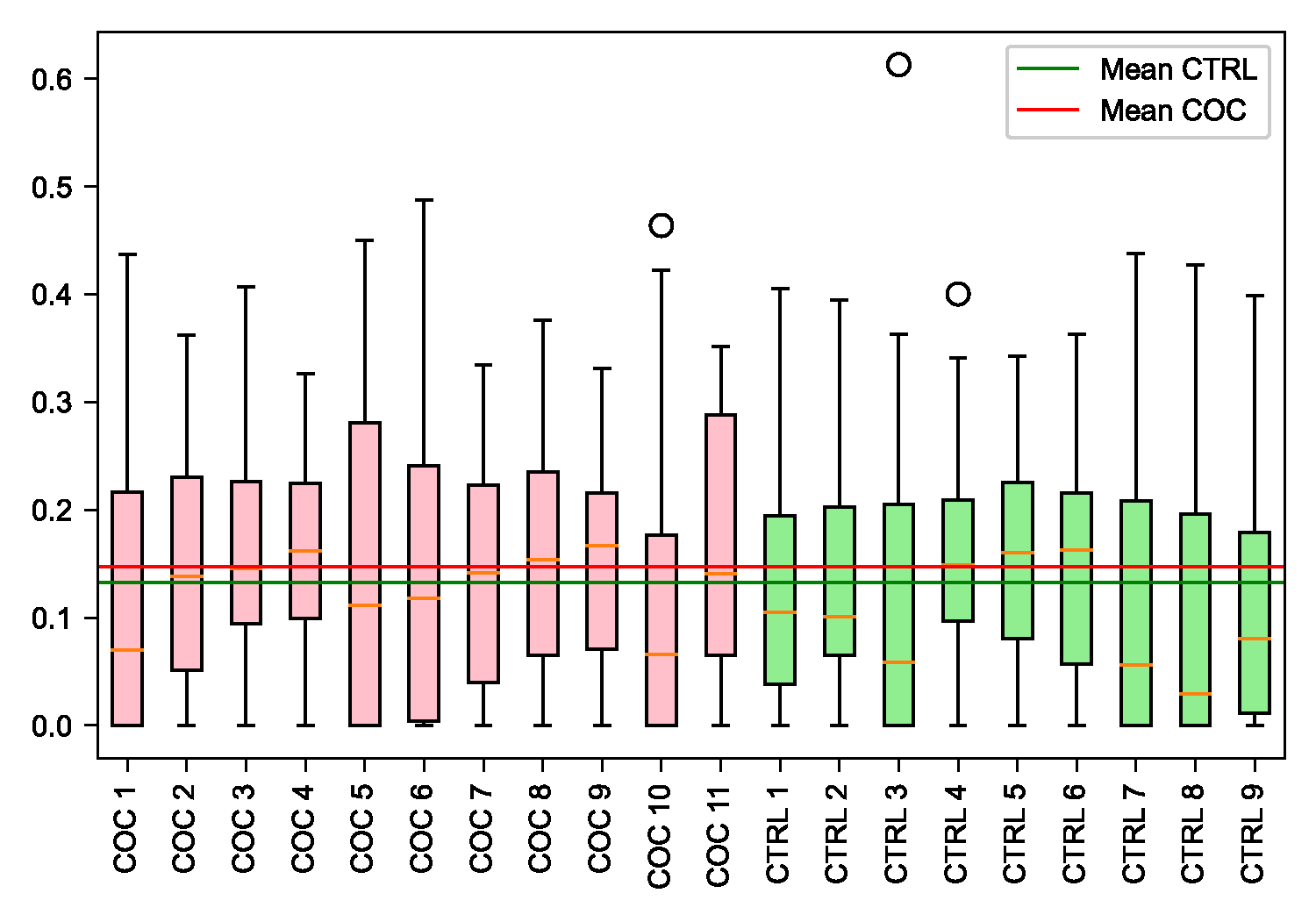

Supplement: S2 Fig — Box plot graph represents measures of Eigenvector centrality measure distribution across networks in CTRL and COC populations. (TIF) [file pone.0275795.s003.tif]

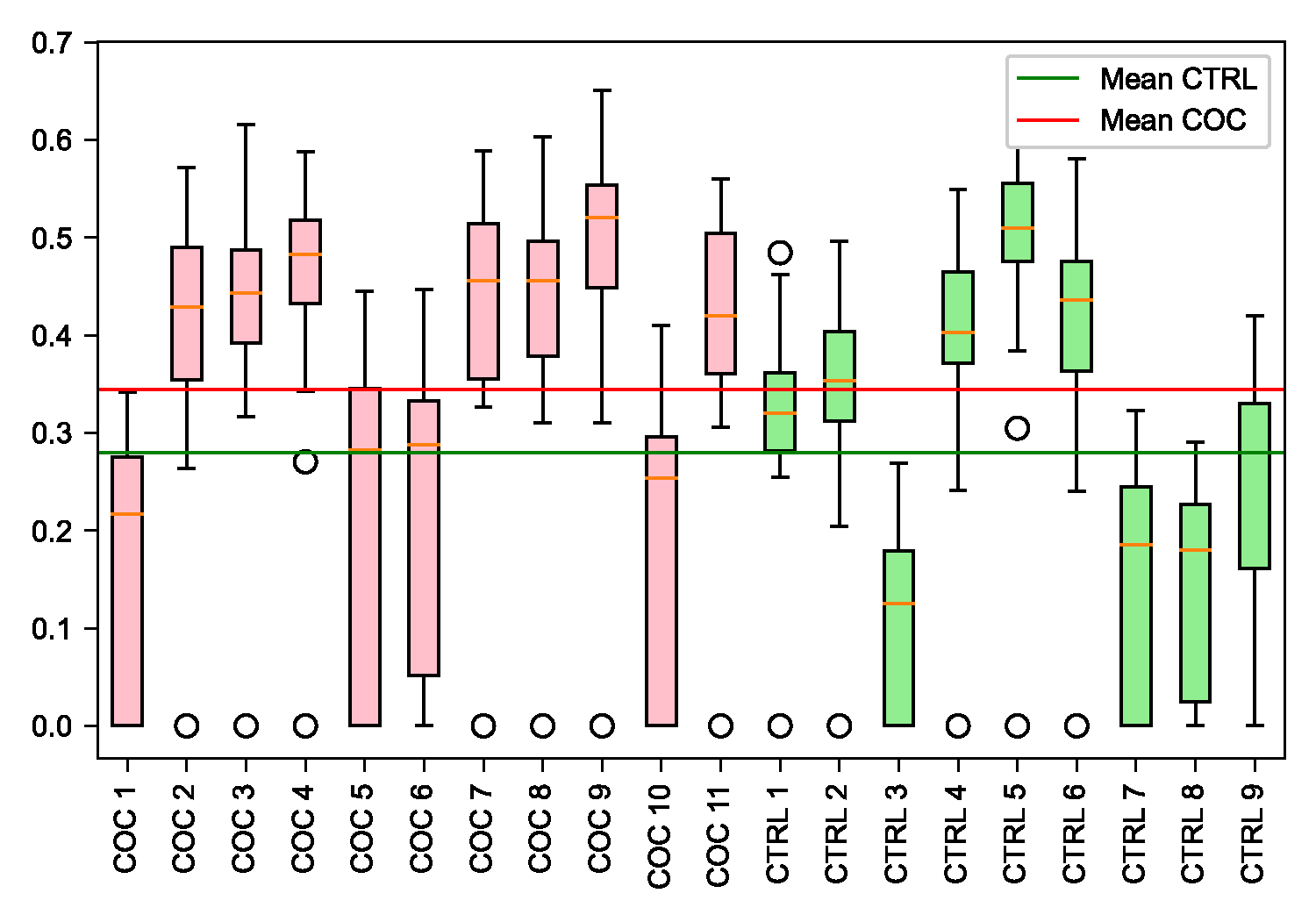

Supplement: S3 Fig — Box plot graph represents measures of Closenes scentrality measure distribution across networks in CTRL and COC populations. (TIF) [file pone.0275795.s004.tif]

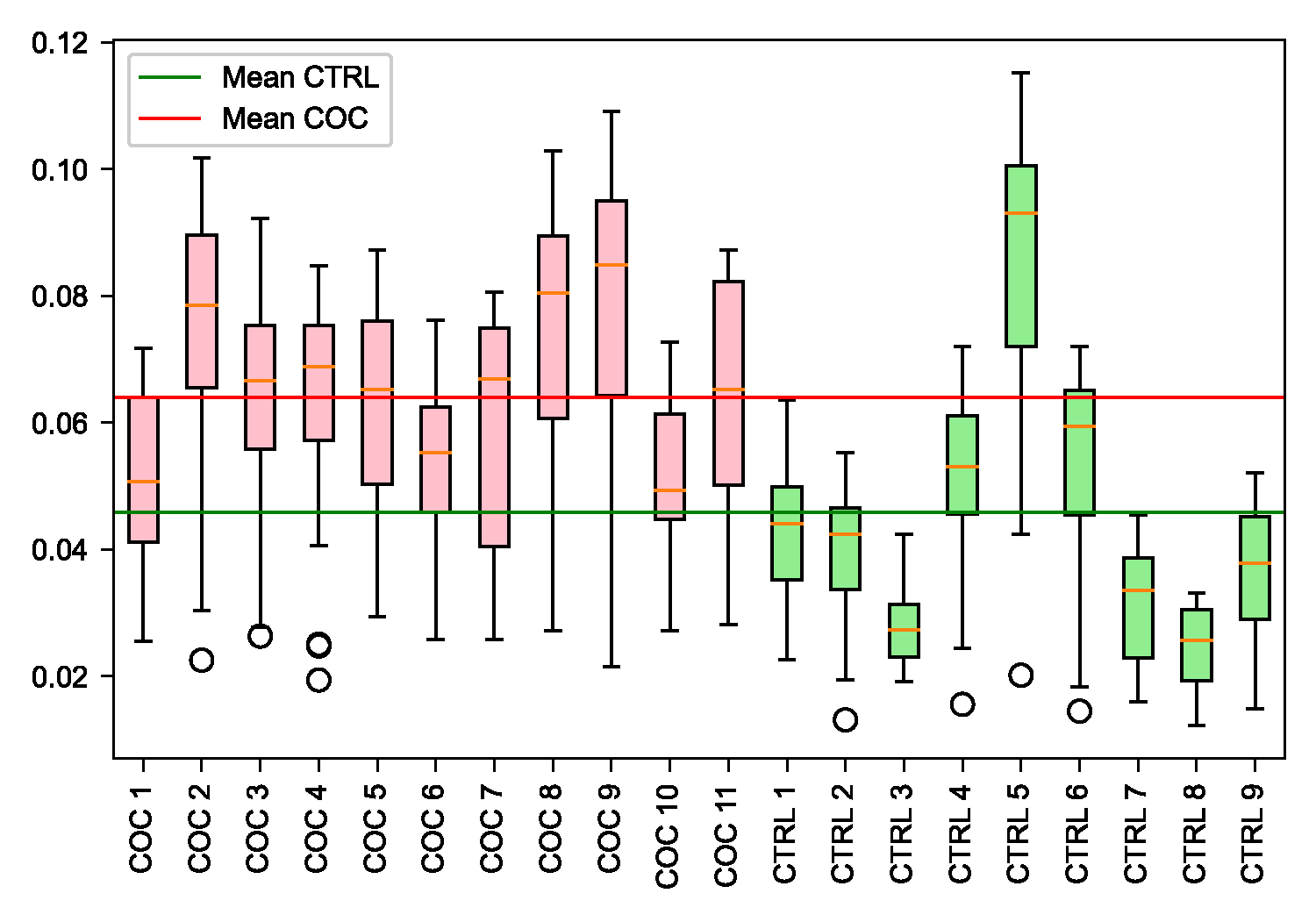

Supplement: S4 Fig — Box plot graph represents measures of Information centrality measure distribution across networks in CTRL and COC populations. (TIF) [file pone.0275795.s005.tif]

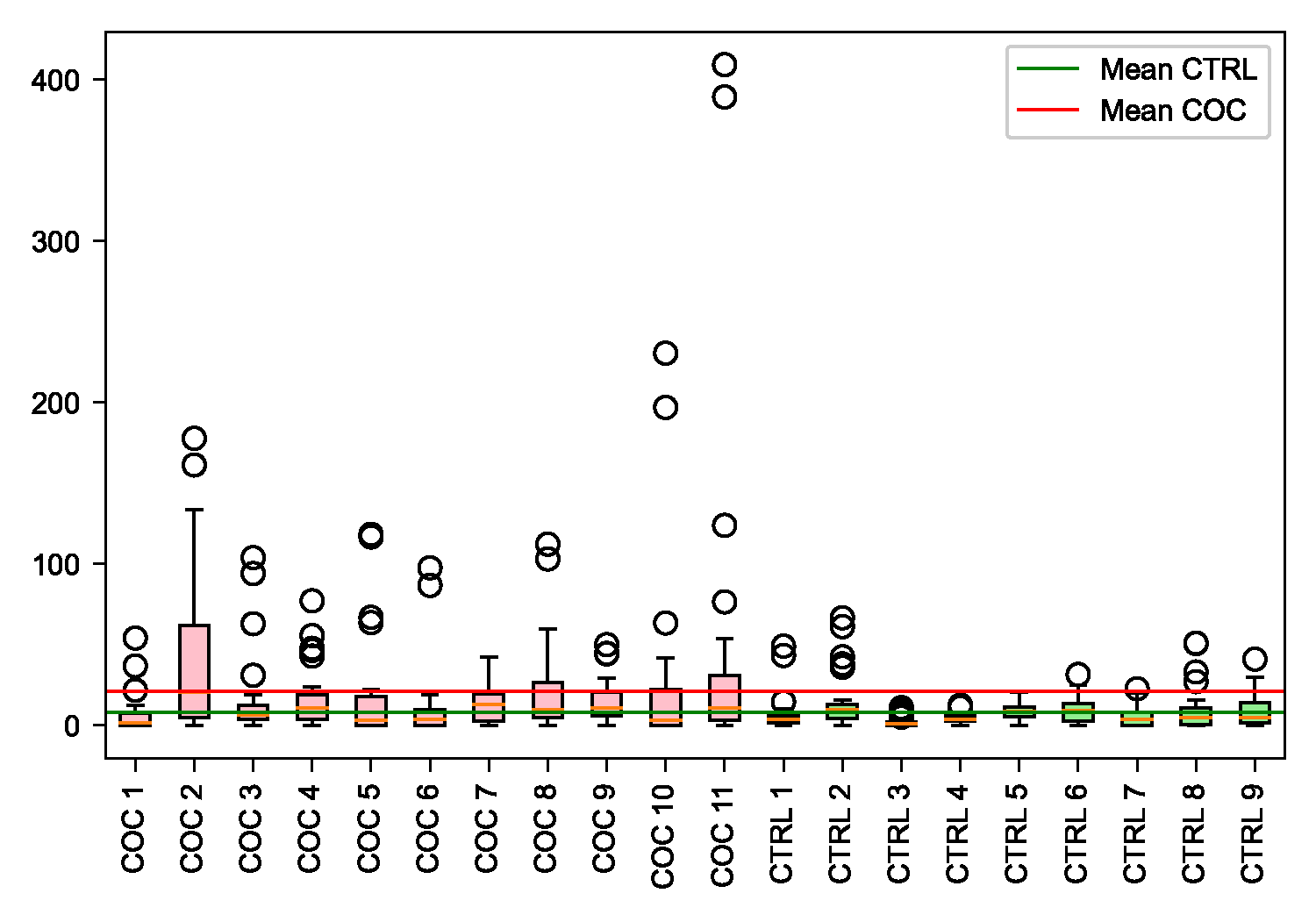

Supplement: S5 Fig — Box plot graph represents measures of Strength measure distribution across networks in CTRL and COC populations, where weight is duration. (TIF) [file pone.0275795.s006.tif]

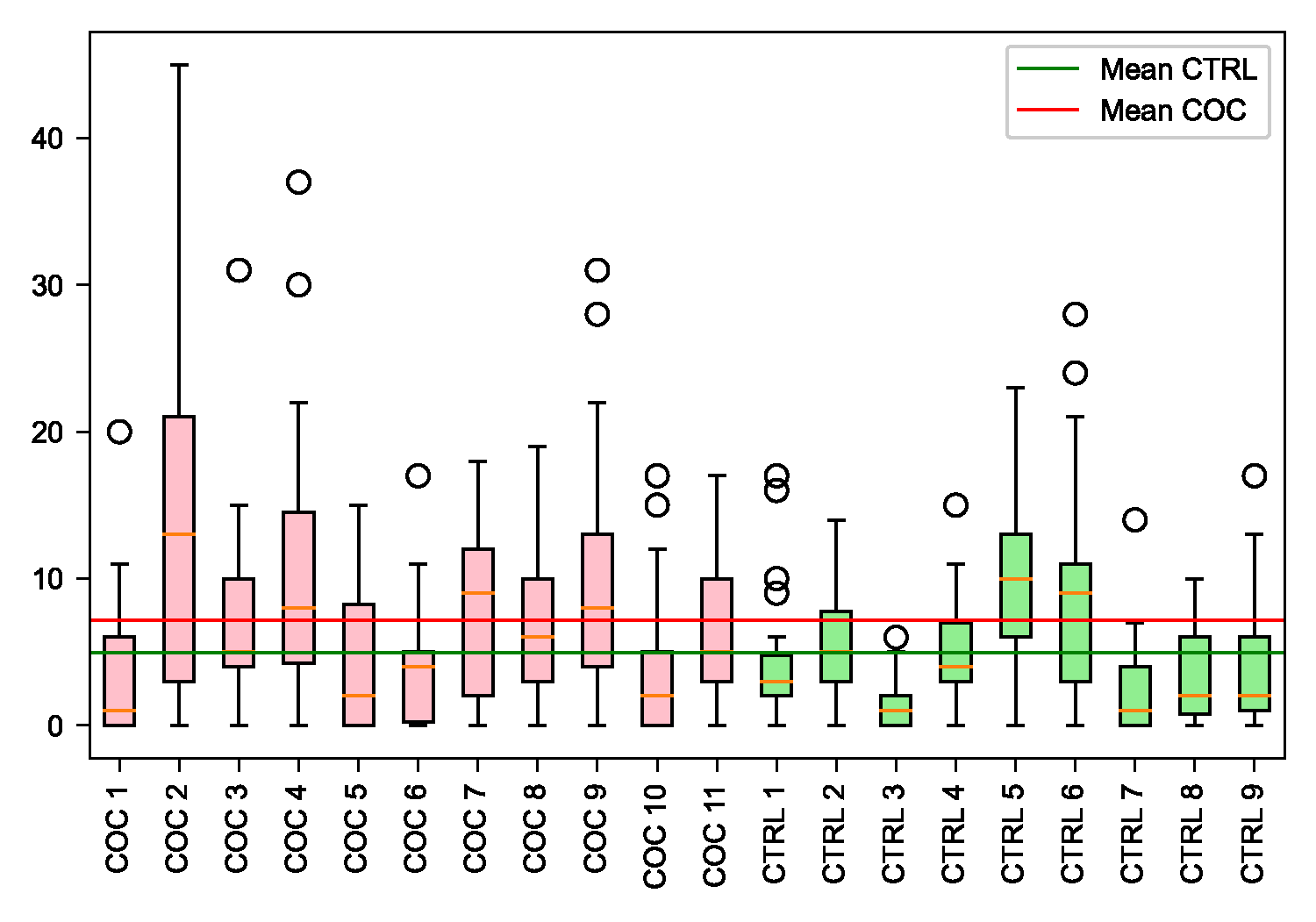

Supplement: S6 Fig — Box plot graph represents measures of Strength measure distribution across networks in CTRL and COC populations, where weight is count. (TIF) [file pone.0275795.s007.tif]

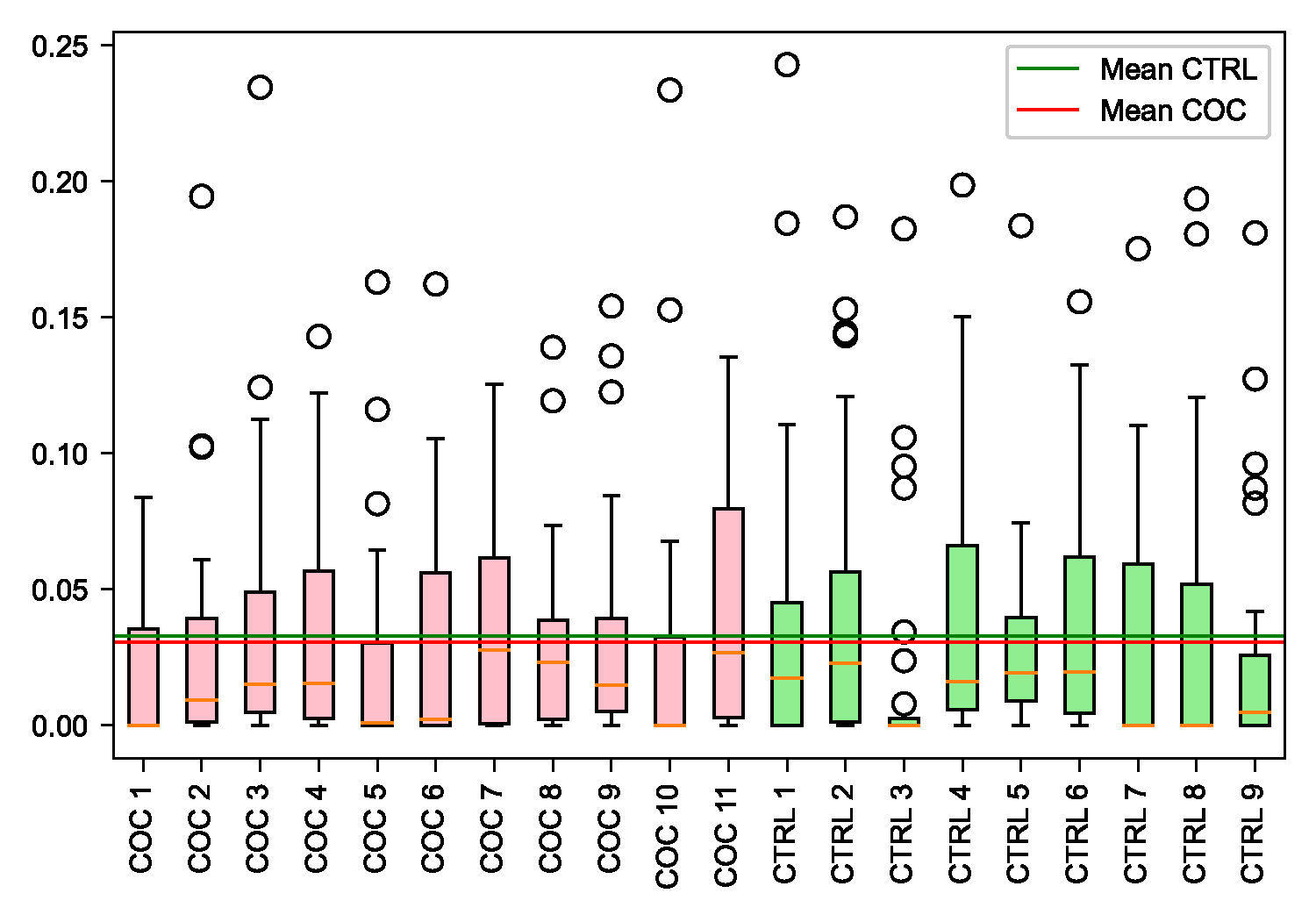

Supplement: S7 Fig — Box plot graph represents measures of Betweenness centrality measure distribution across networks in CTRL and COC populations. (TIF) [file pone.0275795.s008.tif]

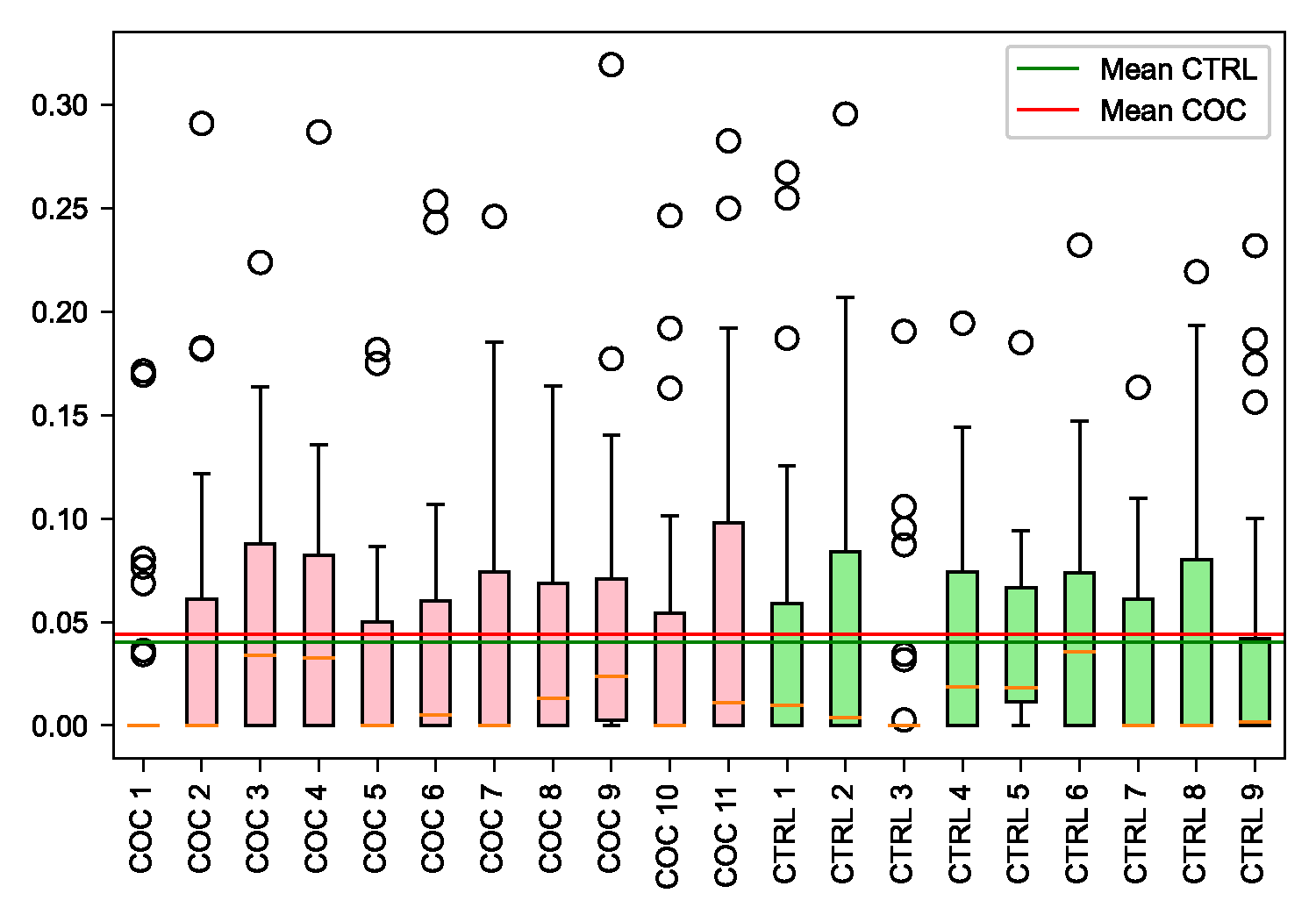

Supplement: S8 Fig — Box plot graph represents measures of Betweenness centrality measure distribution across networks in CTRL and COC populations, where weight is duration. (TIF) [file pone.0275795.s009.tif]

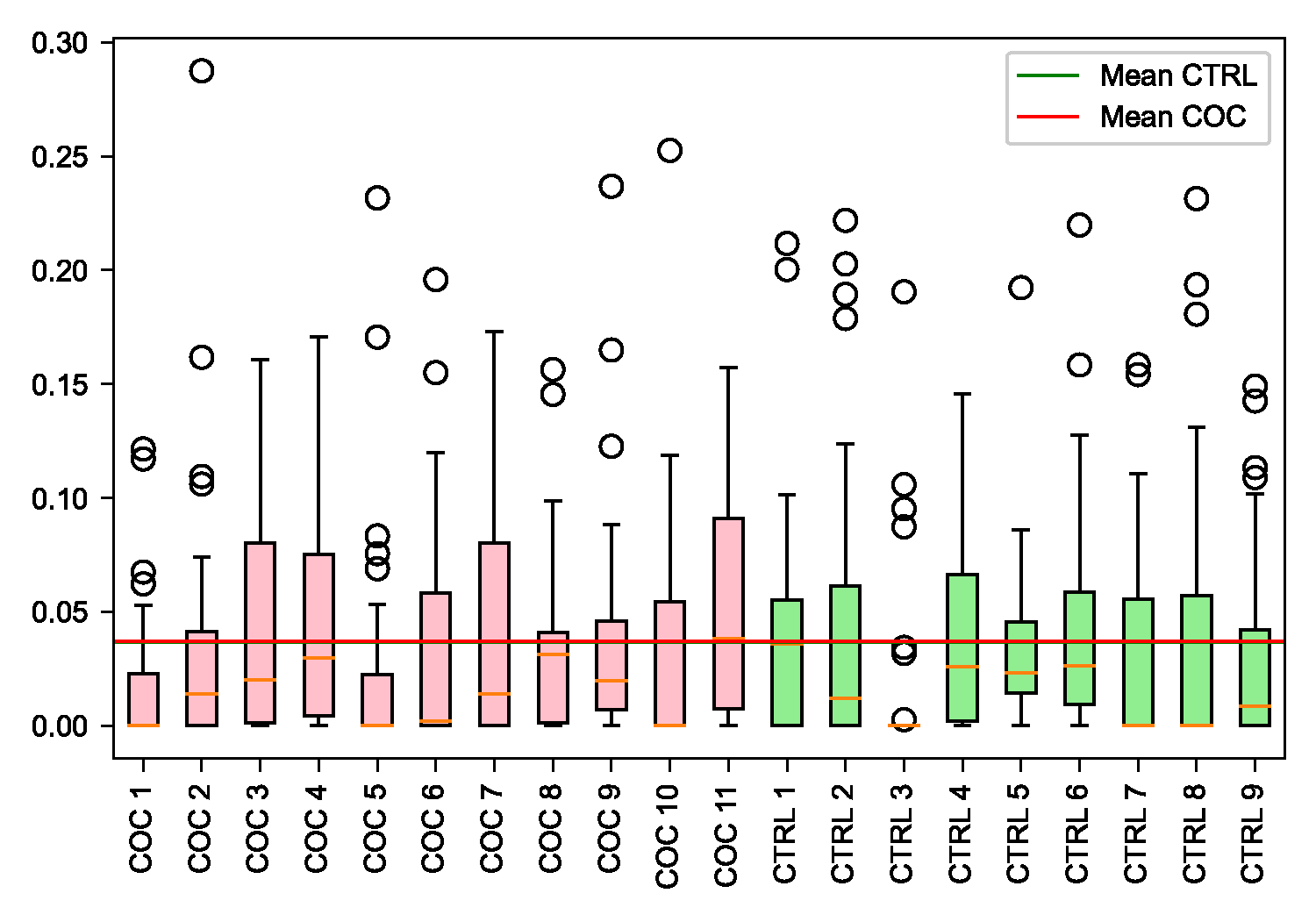

Supplement: S9 Fig — Box plot graph represents measures of Betweenness centrality measure distribution across networks in CTRL and COC populations, where weight is count. (TIF) [file pone.0275795.s010.tif]

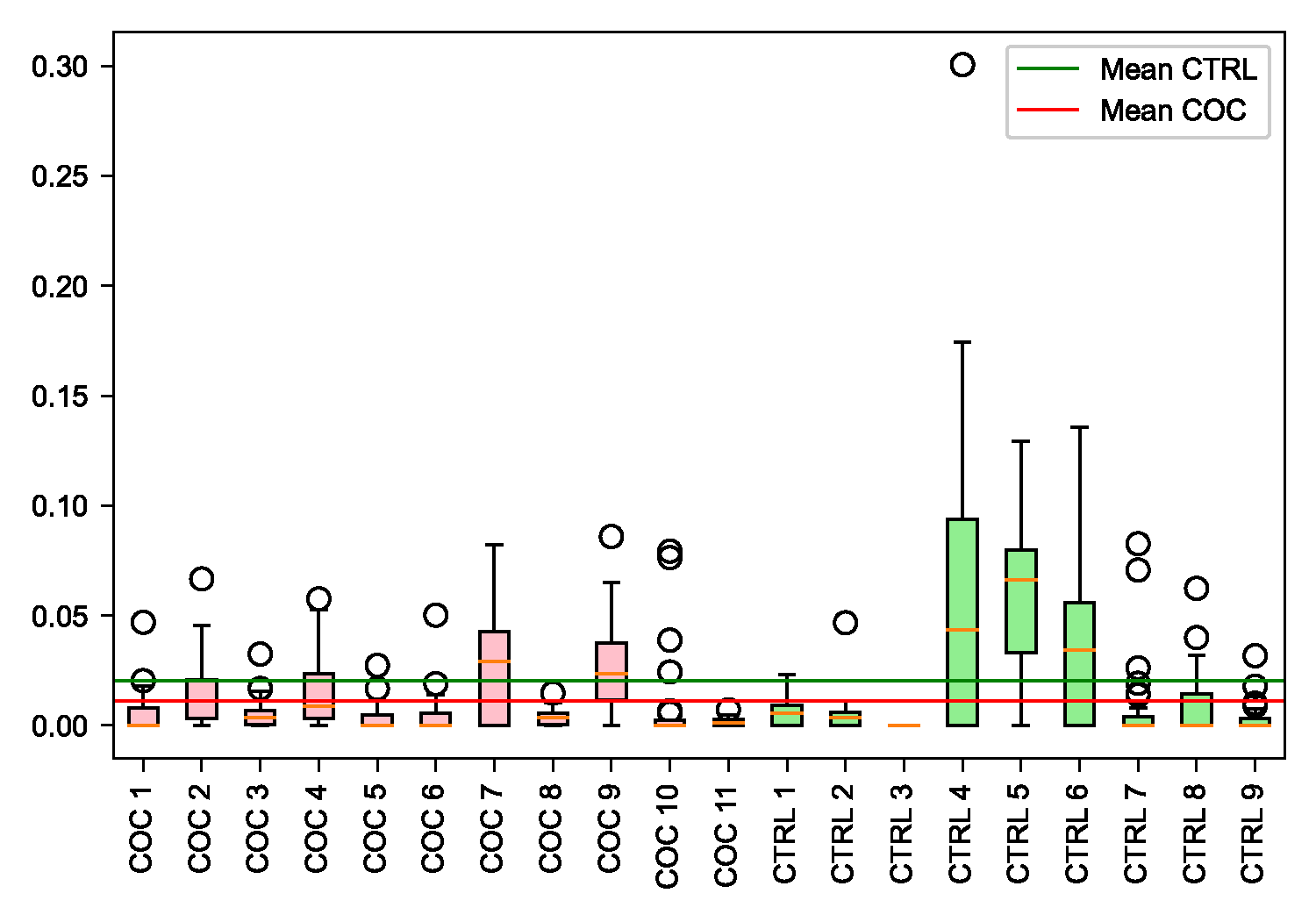

Supplement: S10 Fig — Box plot graph represents measures of Clustering coefficient measure distribution across networks in CTRL and COC populations, where weight is duration. (TIF) [file pone.0275795.s011.tif]

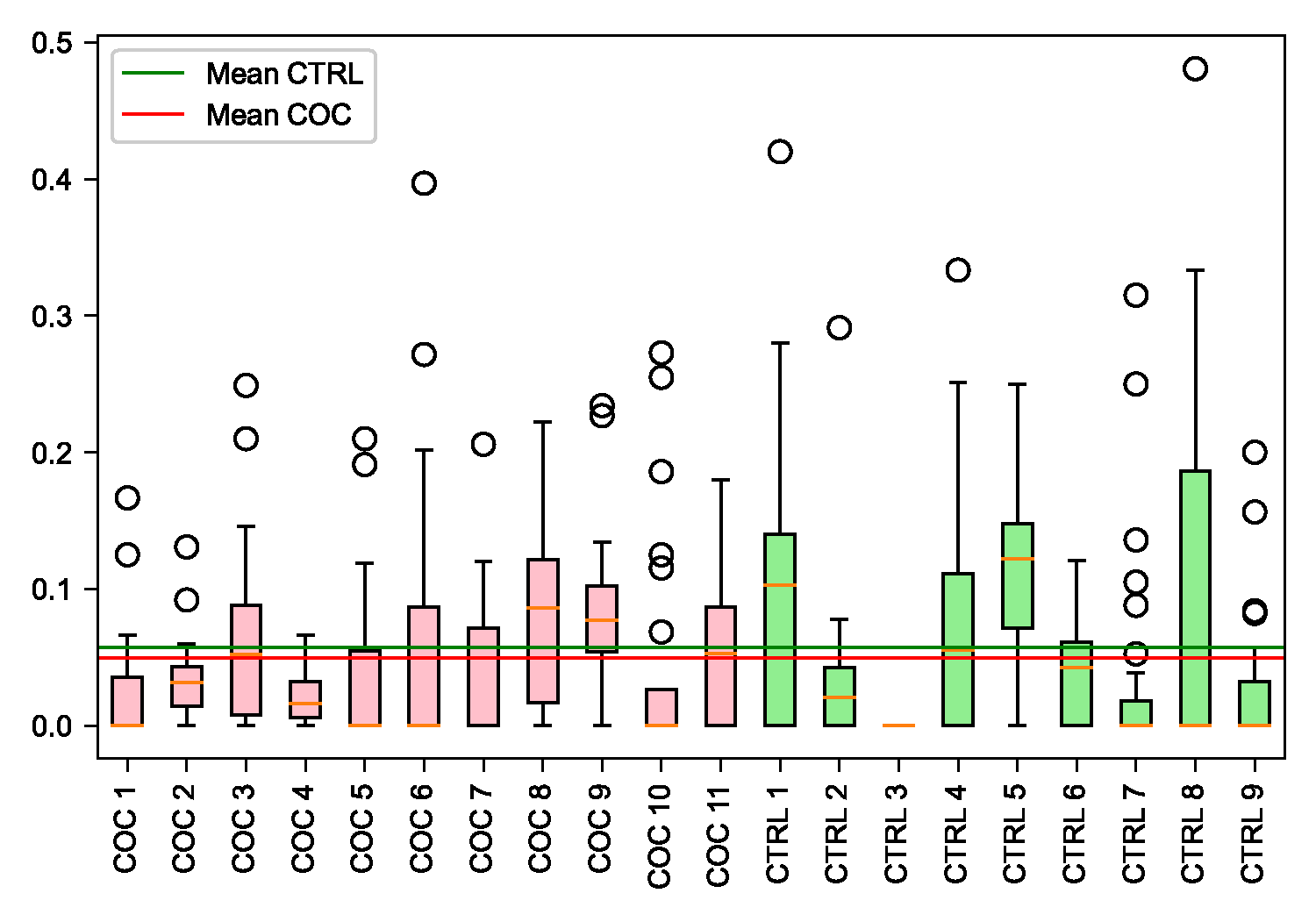

Supplement: S11 Fig — Box plot graph represents measures of Clustering coefficient measure distribution across networks in CTRL and COC populations, where weight is count. (TIF) [file pone.0275795.s012.tif]
