# Supplementary material for: A network-based analysis detects cocaine-induced changes in social interactions in Drosophila melanogaster
Source: PLoS One. 2023 Mar 23;18(3):e0275795. doi: 10.1371/journal.pone.0275795 (PMC10035901; doi:10.1371/journal.pone.0275795)

Rebuttal Letter

We thank the editor and reviewer for detailed reading and for providing suggestions and comments on our manuscript. We are grateful for the opportunity to submit a new version of our paper. Below is our response to each point raised by the academic editor and reviewer. We add some explanations and address all issues and we hope that the manuscript is now suitable for publication. As the academic editor recommended, we deposit our laboratory protocols in protocols.io to enhance the reproducibility of our results

Reviewer:

1. Most of the reviewers' comments were appropriately addressed, and the manuscript is mostly ready for publication. However, I still think that the novelty of the methodology is still not shown. In the introduction, the authors still do not explain why a new methodology is necessary. What is the problem the authors are trying to solve? And in the discussion, it is still unclear to me in what is new and advantageous in the method proposed by the authors compared to what was done before.

Our response. Thank you for this important remark. **We add more details about the novelties of the proposed methodology** in the Introduction and Discussion section and Conclusion as follows.
In the last paragraph of the Introduction section, we now explain that: "*We describe a novel network-based methodology for the analysis of social behavior in adults D. melanogatser. Within the methodology, we propose a set of network-based measures that quantify social interactions of D. melanogaster represented via Social Interaction Networks (SINs). This methodology aims to analyze the behavior induced by psychostimulants by analyzing SINs structures on the local, middle and global levels.*".
And later in the text, we claim that "*To the best of our knowledge, this is the first attempt to apply network-based analysis at the local, middle and global level to detect cocaine-induced changes in Drosophila social interaction networks.*"
Thus, the main novelty and advantages of the proposed methodology are in the selected set of the local, middle and global network measures which have never before been used to analyse *Drosophila* social interaction networks.
We also explain in the Introduction section what can be analysed using the proposed method:
The methodology enables "*identifying structural properties and differences in D. melanogaster populations. The proposed approach provides insight into parameters and characteristics of group behavior in the presence and absence of psychostimulants that could be of interest for further bioinformatics research studies and analyses.*"
We add a more detailed explanation in the discussion section:
„ *Within the proposed methodology, we select and calculate a set of network-based measures that provide insight into the network structure and possibly behavioral properties of files represented as nodes within the observed SINs. Specifically, we showed that selected network measures can differentiate between COC and CTRL Drosophila melanogaster populations. We implemented the proposed methodology using NetworkX and utilized this approach to detect cocaine-induced changes in social interactions in Drosophila melanogaster.*"

1. Does NetworkX lead to less errors than Ctrax? Is it easier to use? Does it allow more levels of analysis? A direct clear comparison is necessary.

Our response. We agree that this may not be clear in the previous version of our manuscript, but as you noticed, NetworkX actually enables more levels of analysis while Ctrax is software for tracking the

flies from video files. We stated that the Ctrax tool was originally used for tracking flies in videos. After that, we stated that the Flytracker tool is more precise, that it has a much smaller chance of making an error in the tracked videos. And that is the reason of using Flytracker software.

Now we explain that more clearly:

„Although often used, Ctrax can lead to errors when tracking individuals, due to the loss of tracking or swapping of intensities, so new software has been developed that eliminates these problems.“

“*We used Flytracker for tracking the flies in the first step of our experiment. In the next step we used a library called NetworkX, written in the Python programming language, and implement all data processing and network-based analysis. Thus we were able to automatically extract and analyze SINs data from 10 minute videos of 30 individuals \cite{alisch_maple_2018}. For network visualisations we used Gephi, an open source software \cite{gephisoftware}.*”

And we add Conclusion section we now explain that “*We implement the methodology using NetworkX library enabling the efficient analysis of the network structure at local, middle and global level. Implementation of the proposed approach in NetworkX provides a novel level of analysis and insight into social interactions of D. melanogaster.*”

2. Edits: I suggest running a spelling and grammar check before final submission, as I picked some typos in the abstract and introduction, but stopped checking there. ,

Third sentence abstract: “we have applied local, middle and global level”: add comma after “middle”

Firth sentence: “and formed larger, densely populated and compact communities” : add comma after “compact”

Last sentence of abstract: Our approach can be expandED on

L2: I propose: “Drug addiction is a complex…”

L6: remove the comma after “abuse”

L10: “This events perturb” --> these events perturb

L16: “genES pleiotropy” and “”difficult to assignee”

L20: “metric propensity” --> propensities

L25: “This approach maskS”

L32: “transLational

L40: “neuronal citrus analysis” ⍰ what do you really want to say?

L75: “by using A library…”

L493: reference missing

Our response. Thank you for reading our manuscript in detail and help us with these remarks.

We sent the paper to proofreading and correct all language mistakes. We added the missing reference.

Thank you once again for all suggestions.

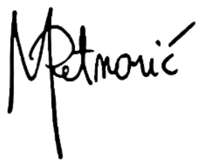

Supplement: Attachment — Submitted filename: response_to_reviewers.pdf [file pone.0275795.s020.pdf]
